# Evaluation of results obtained with corifollitropin alfa after poor ovarian response in previous cycle using recombinant follicular stimulating hormone in the long-term protocol

**DOI:** 10.5935/1518-0557.20160028

**Published:** 2016

**Authors:** Lister L. Salgueiro, Juliana R. Rolim, Bernardo R. L. Moura, Suelen P. P. Machado, Carolina Haddad

**Affiliations:** 1 Clínica Fértilis de Reprodução Assistida, Sorocaba, SP, Brazil

**Keywords:** In vitro fertilization, Poor responders, Corifollitropin alfa

## Abstract

**Objective:**

This study evaluated the use of Corifollitropin alfa in patients with previous poor response to recombinant follicle stimulating hormone in long-term protocols using gonadotropin-releasing hormone.

**Methods:**

Twenty-seven poor responders to previous treatment with the long term protocol using the recombinant follicle stimulating hormone (Group 1) were selected and then submitted to a second attempt using the same long term protocol with Corifollitropin alfa instead of the recombinant follicle stimulating hormone (Group 2).

Ovarian down-regulation was achieved using subcutaneous administration of Leuprolide Acetate. Ovarian stimulation was performed with recombinant follicle stimulating hormone until the administration of human chorionic gonadotropin, followed by follicular aspiration (Group 1). Group 2 was submitted to this same protocol using Corifollitropin alfa instead of recombinant follicle stimulating hormone.

**Results:**

There were significant differences in the number of aspirated oocytes, percentage of mature oocytes, amount of injected oocytes and transferred embryos - with all of these parameters being increased in the Corifollitropin alfa group. In addition, the rates of pregnancy and ongoing pregnancy were also significantly higher in the Corifollitropin alfa group.

**Conclusion:**

The present study demonstrated that the use of Corifollitropin alfa in the long-term protocol could be a highly effective alternative for patients with poor ovarian response, who were unsuccessful in a previous treatment with In Vitro Fertilization - Intracytoplasmic Sperm Injection.

## INTRODUCTION

Historically, In Vitro Fertilization (IVF) treatment in poor responder patients has been a great challenge and the object of several published studies. Despite the large amount of data found in literature regarding the management of these patients, none of the treatments available has been adopted as default, due to inconsistencies concerning the significant benefits. Therefore, the identification of a new treatment option for these women seems to be crucial (Polyzos *et al*., 2013).

Recently, a new molecule from a long-term recombinant gonadotropin has been introduced in clinical practice with pregnancy rates similar to known treatments with recombinant follicle stimulating hormone (rFSH). Corifollitropin alfa (CA) is a recombinant hormone consisting of an alpha subunit, identical to FSH, and a beta subunit - produced by the fusion between the C-terminal peptide from the beta subunit of human chorionic gonadotropin (hCG) and the beta subunit of FSH. A single injection of CA is capable of initiating follicular growth and sustaining it for a week with a slow absorption reaching maximal concentrations two days after the injection and declining progressively afterwards (Bouloux *et al*., 2001; Duijkers *et al*., 2002; Fauser *et al*., 2009; 2010; Devroey *et al*., 2004). Previous studies revealed that 100 mcg of CA is the optimal dose for women up to 60 kg and 150 mcg for women over 60 kg (Devroey *et al*., 2009; The Corifollitropin alfa ENSURE study group, 2010), initial studies demonstrated that the use of CA is highly effective and safe for ovarian stimulation, when used with Gonadotropin-Releasing Hormone (GnRH) antagonist (Kolibianakis *et al*., 2006; Mahmoud Youssef *et al*., 2012). This activity, when used with the long-term protocol could be considered a risk factor for Ovarian Hyperstimulation Syndrome (OHSS). Due to this the use of CA with the long-term protocol could be a drug of choice in the case of poor responders or in women of advanced age.

In the present study, we used CA in patients with poor response to the first IVF cycle using long-term protocol (GnRH agonist) and rFSH.

## MATERIALS AND METHODS

The study This retrospective study included twenty-seven patients submitted to In Vitro Fertilization (IVF) treatment. All patients that poorly responded to previous treatment with the long-term protocol using rFSH (rFSH - Gonal-f^®^ Merck Serono, São Paulo Brazil) (Group 1), and then were submitted to a second attempt using the same long-term protocol with CA (Elonva^®^, MSD, São Paulo - Brazil) (Group 2).

### Down-Regulation

The protocol was initiated on the 21st day of the previous menstrual cycle, with subcutaneous administration of 0.1 ml/day of Leuprolide Acetate (Lupron® kit - Abbott - Brazil), for down-regulation of the gonadal axis. After 14 days, serum levels of estradiol (E2) were measured and transvaginal ultrasound (TVUS) was performed. Down-regulation was confirmed by the presence of menstruation, E2 serum levels lower than 50 mUI/mL and TVUS without follicles bigger than 20 mm. After down-regulation confirmation, the ovarian stimulation was initiated.

### Group 1 (G1)

Leuprolide acetate was maintained in the same dose, and ovarian stimulation was initiated with rFSH. Patients up to 30 years of age received FSHr, 150 UI/day in the first seven days, with a reduction (step-down) to 75 UI/day in the following days until the day of hCG (Ovidrel^®^ Merck Serono, São Paulo Brazil) administration. Patients between 30-37 years of age used 225 UI/day in the first seven days, followed by stepping it down to 175 and 75 UI/day in the following days until hCG administration. Patients over 37 years of age received 300 UI/day in the first seven days, with step-down to 225, 150, and 75 UI/day in the following days until hCG administration.

The first ovarian control was performed by TVUS on the seventh day of stimulation to follow follicular growth (amount and average diameter) and endometrium assessment (diameter and classification).

The second control by TVUS was performed on the tenth day and on hCG administration date, scheduled according to follicular growth speed (at least two follicles with 20-22 mm). Follicular aspiration was performed 35 hours after hCG administration.

### Group 2 (G2)

In the second cycle (G2), the same patients, who responded poorly and did not achieve pregnancy, were submitted to a new Controlled Ovarian Hyperstimulation (COH), using the same long-term down-regulation protocol; however, using CA instead of rFSH. Patients weighing 60kg or less received 100 mcg of CA, and patients over 60kg received 150 mcg of CA.

Seven days after stimulation onset, the first ultrasound control was performed to follow follicular growth and endometrial quality. From that day on, the patients were given a complement of 75 UI/day of rFSH until the day before hCG administration.

The second control was performed on the tenth day, and the aspiration date was stipulated according to follicular growth (at least two follicles with 20-22 mm). Follicle aspiration was then performed 35 hours after hCG administration.

### Laboratory work up and pregnancy

Laboratorial parameters were the same for both groups, with no differences in sample manipulation and embryonic cultivation, performed by the same embryologist - therefore discarding any possible variables in this aspect.

In both groups, the transfer of embryos was performed on day three, selecting the best embryos. After twelve days of transfer, pregnancy was confirmed through the serum levels of human chorionic gonadotropin (beta-hCG).

### Data analysis

Statistical significance was determined by the Wilcoxon test (paired samples), t-Student (paired samples) and McNemar tests. Differences were considered significant for a *P* value ≤ 0.05.

### Institutional Review Board

It was not required because this is a study carried out exclusively from patient files, with no handling of biological material from patients and without disclosing patients' information.

## RESULTS

The Data from 27 patients submitted to in vitro IVF-ICSI cycles between 32 and 41 years of age were analyzed and are presented on Table 1.

**Table 1:** Comparison between two cycles from the same patient using different medications.

	G1 n=27 Mean±SD	G2 n=27 %	Mean±SD	%	*P* value
Average Age	36.9 ± 3.4		37.01 ± 3.4		0.022*
Average number of oocytes	2.8 ± 1.6		5.1 ± 2.1		<0.001
MII Oocytes (%)	2.81 ±1.56	63.3 ± 30.8	3.85 ± 1.81	75.2 ± 16.4	0.109
Injected	1.8 ±1.2		3.9 ± 1.8		<0.001
Fertilized					
2PN (%)	1.70 ±1.17	94.7 ± 14	3.51 ± 1.64	92.9 ± 11.8	0.442
Cleaved (%)	1.70 ±1.17	100 ± 0	3.51 ± 1.64	100 ± 0	>0.999
Embryo Quality in 72 hours A+B (%)	1.37 ± 1.50	83.2 ± 21.8	2.51 ± 1.38	77.1 ± 23.3	--0.107
C+D (%)	0.33 ± 0.65	41.1 ± 11.3	1 ± 1.55	38.8 ± 22.3	0.779
ET Average	1.6 ±1		2.6 ± 0.7		<0,001
PR	0		12	44.4%	0.001**
Ongoing	0		11	40.7%	0.002**

Wilcoxon test;

* t-Student test;

** McNemar test

MII oocytes: metaphase II oocytes; 2PN: oocytes showing two pronuclei; A+B: quality embryos; C+D: quality embryos. ET: transferred embryos; PR: Pregnancy Rate; G1: first cycle (using Gonal); G2: second cycle (using Elonva).

### Laboratorial and Clinical Aspects

According to Table 1, several parameters were analyzed and compared between the groups. There was a significant difference (*P*=0.022) regarding the average age (36.9 ± 3.4 and 37.01 ± 3.4) between groups 1 and 2, because they were the same patients but in different cycles. The difference in the average number of aspirated oocytes was statistically significant (*P* < 0.001), 2.8 ± 1.6 in G1 and 5.1 ± 2.1 in G2. Regarding the percentage of mature oocytes (MII), no significant difference was found between the groups (*P*=0.109), G1 with 63.3 ± 30.8% and G2 with 75.2 ± 16,4%. The difference in the amount of injected oocytes was also significant (*P* <0.001) G1: 1.8 ±1.2, G2: 3.9 ± 1.8. Fertilization and cleavage did not show statistical differences between the cycles (*P*=0.442 and >0.999). After 72 hours, the percentage of embryos A+B was lower in the second cycle, but no significant difference was found (83.2 ± 21.8 and 77.1 ± 23.3; *P*=0.107). The average of transferred embryos per patient was significant, with 1.6 ± 1 in G1 and 2.6 ± 0.7 in G2 (*P*<0.001).

### Pregnancy

The rate of pregnancy was statistically different and significant between the groups, going from 0% in the first cycle to 44.4% in the second cycle; the percentage for ongoing pregnancy was 0% in G1 and 40.7% in G2 bearing statistical significance.

## DISCUSSION

In this study we comparatively evaluated rFSH and Corifollitropin alfa in different cycles of ovarian stimulation of a same patient, considered poor responder and with normal base levels of FSH, in the long-term protocol using the association between Elonva and Gonal-F.

Studies evaluating ovarian response, in protocols with GnRH agonists and antagonists, reported higher follicular recruitment when CA was used (Fluker *et al*., 2001; Andersen *et al*., 2006). These studies suggest that after profound suppression, CA recruits a higher cohort of follicles when compared to daily rFSH, due to increased circulation of FSH during the first days of stimulation (Duijkers *et al*., 2002). On the other hand, according to Polyzos *et al*. (2013) when comparing cycles with CA and cycles after short-term protocol with GnRH agonist, the cohort of follicles found was equivalent.

In the present study, there was a significant improvement after CA use in those patients that did not respond well to the first treatment. Studies using GnRH antagonist demonstrated the efficacy of this medication when used in patients presenting with poor ovarian response (Devroey *et al*., 2004).

The results observed in our study corroborate the meta-analysis performed by Mahmoud Youssef *et al.* (2012), where they reported a significantly higher number of MII oocytes in the group that received CA compared with rFSH. In addition, in this same study, there was no significant difference in fertilization rates between the groups. However, in the present study we found a higher number of embryos produced in the CA group in comparison with the rFSH group, which does not agree with the study by Mahmoud Youssef *et al.* (2012). We believe that this difference in the number of embryos is related to a significant difference in mature oocytes between the groups.

Regarding rates of fertilization, cleavage and embryo quality, there was no significant difference between the groups, suggesting that both drugs do not directly influence these parameters.

Most comparative studies report no significant difference in pregnancy rates and ongoing between the groups. However, most of those studies are double blind and randomized and, therefore, do not characterize the individuality of each patient's response. In our study we demonstrate a significant difference in both pregnancy and ongoing (42.86%; *P* =0.031), prioritizing CA. According to van Wely *et al*. (2011) and Croxtall & McKeage (2011), CA is not normally used in long-term protocols with agonist for the large amount of follicles expected, for high risk of ovarian hyperstimulation and consequent cycle cancellation. Nevertheless, in the present study, the patients coming from a long term cycle with rFSH presenting poor response and then submitted to a second cycle, using CA also in a long term protocol, did not present OHSS. It is difficult to estimate the risk of developing OHSS in the absence of known predisposing factors. However, the use of long term protocol, with association between CA and daily rFSH, was shown to be safe for poor responder patients in a previous cycle.

The present study demonstrated that the use of Corifollitropin alfa associated to daily rFSH in the long term protocol could be a highly effective alternative for patients with poor ovarian response that did not obtain success in a previous treatment with IVF-ICSI.

In the next phase of this study we will evaluate the comparison between two random groups, one with patients repeating the same first protocol with FSHr, and group two with patients using Corifollitropin after FSHr use.
